# Antiaging, Brightening, and Antioxidant Efficacy of Fermented Bilberry Extract (*Vaccinium myrtillus*): A Randomized, Double-Blind, Placebo-Controlled Trial

**DOI:** 10.3390/nu16142203

**Published:** 2024-07-10

**Authors:** Vincenzo Nobile, Stéphanie Dudonné, Catherine Kern, Gloria Roveda, Christine Garcia

**Affiliations:** 1 R&D Department, Complife Italia S.r.l., 27028 San Martino Siccomario, Italy; vincenzo.nobile@complifegroup.com; 2 Seppic Research and Innovation, 92250 La Garenne Colombes, France; 3 Clinical Trial Department, Complife Italia S.r.l., 27028 San Martino Siccomario, Italy

**Keywords:** bilberry extract, polyphenols, skin aging, skin complexion, clinical trial, nutricosmetics

## Abstract

Strategies for successful aging, including the use of food supplements, are part of the approach to support skin youthfulness. To demonstrate the efficacy of fermented bilberry extract (FBE) against skin aging and uneven complexion, a clinical trial was carried out on 66 subjects with visible “crow’s feet” wrinkles, mild-to-moderate skin slackness, and uneven skin tone. The wrinkle depth, skin smoothness (Ra) and roughness (Rz), skin firmness (R0) and elasticity (R2), skin coloration (ITA°), and skin antioxidant capacity were measured before and after 28 (D28), 56 (D56), and 84 (D84) days of product use (either FBE or a placebo). These parameters were also integrated with a clinical evaluation, carried out by a dermatologist, and a self-assessment questionnaire to align the measured efficacy with the visual or perceived efficacy. At D84, the wrinkle depth had decreased by 10.6%, Ra had improved by 7.9%, Rz had decreased by 7.3%, R0 had improved by 13.3%, R2 had improved by 12.4%, and skin antioxidant capacity had increased by 20.8%. ITA° increased by 20.8% and was accompanied by a decrease in the skin’s redness component by 16.8% and an increase in the lightness component by 2.2%. The variation of all the above-mentioned parameters was statistically significant between the FBE and PL groups. Our findings demonstrate the efficacy of FBE in improving skin aging and complexion evenness.

## 1. Introduction

The skin is a complex multilayer organ that performs important functions in separating and protecting the body from the environment (i.e., pathogenic microbes, chemical agents, and physical factors), maintaining electrolyte homeostasis, preventing enhanced water loss, thermoregulation, and immune response [1,2].

Skin aging is part of the natural “aging mosaic” and is driven by both intrinsic (genetic and chronological) and extrinsic (exposome) factors [3,4,5,6]. Both factors induce cellular senescence and are associated with increased oxidative stress [7,8,9,10]. Alterations in the skin structure, metabolism, and physiology due to aging change the skin aspect [11]. The clinical features that are characteristic of aged skin include skin wrinkling and alteration of the skin’s texture, dryness, loss of elasticity, uneven pigmentation, discoloration, alteration of skin complexion, telangiectasias, and sensitivity [1,12,13,14]. Even if this condition is not life-threatening, the alteration of the skin’s appearance has an important emotional and psychological impact influencing the subject’s self-esteem and social relationships [15]. The aged skin’s appearance can also alter how individuals are seen by others due to the misleading interpretation of character traits [16] or emotions (e.g., sadness, tiredness, etc.) that do not correspond to the true feelings of the individual [17,18].

Historically, the treatment options for skin aging were considered the purview of the cosmetic industry or plastic surgery. However, in recent years, there is increasing evidence in the scientific literature of a positive link between a balanced diet and the appearance and functioning of the skin [19,20,21]. This awareness has increased the demand for food supplements that can improve the skin from inside to give beauty outside, i.e., “nutricosmetics” [22]. Among them, food supplements enriched with antioxidants, especially polyphenols, have been shown to have a beneficial effect on skin aging [1,23,24,25,26,27].

In this trial, we aimed to investigate the efficacy of fermented bilberry (*Vaccinium myrtillus*) extract (FBE) in reducing the clinical signs of skin aging and improving skin tone evenness. Bilberry (or European blueberry) is a dwarf shrub native to northern Europe but is now also found in parts of North America and Asia [28]. Bilberries have a long-standing tradition of use in treating a wide range of disorders and have marketing claims of being a “superfood” or a “functional food” [29,30,31,32,33,34]. Bilberry bioactive compounds include anthocyanins (delphinidin and cyanidin), flavanols (catechin and epicatechin) and proanthocyanidins, flavonols (quercetin and myricetin), phenolic acids, and terpenoids (triterpenoids, tetraterpenes, and iridoids) [34]. Fermentation is considered to be an efficient method to overcome the poor bioavailability of the above-mentioned berry polyphenols in the gastrointestinal tract [35,36] and to increase their functional properties, as demonstrated by the latest scientific evidence [37]. 

The study protocol of the present trial was designed based on the results of a pilot study carried out previously (data not published) to further investigate the antiaging efficacy of the extract, along with its efficacy for the skin and complexion (brown and red components) and in improving the skin’s antioxidant capacity.

## 2. Materials and Methods

### 2.1. Trial Design

This was a single-center, randomized, placebo-controlled, parallel-group trial conducted in Italy at the Complife Italia S.r.l. facilities (Italy) between July 2023 and January 2024. Each subject attended a clinic visit at screening, at baseline (D0), and after 28 (D28), 56 (D56), and 84 (D84) days of product intake. After enrolment, subjects were randomly assigned to the active (FBE) and the placebo group (PL) at baseline. 

The trial was carried out according to the study protocol (ref. no. 2023/08 on 2 August 2023) approved by the “Comitato Etico Indipendente per le Indagini Cliniche Non Farmacologiche” (Genova, Italy) and in full accordance with the principles of the Declaration of Helsinki and its amendments. The clinical trial was registered at www.clinicaltrials.gov (NCT06032598).

### 2.2. Interventions and Randomization

The active treatment arm of the cohort (FBE) received 1 capsule (in the morning at breakfast) containing 100 mg of fermented bilberry extract (Sepitone^TM^, Seppic, La Garenne Colombes, France), 335 mg of maltodextrin, and 10 mg of magnesium stearate. FBE is obtained from fermenting wild bilberry fruit with wine yeast (*Saccharomyces cerevisiae*). The fermentation medium is filtered and concentrated, then purified by precipitation with hot ethanol, and finally spray-dried [38]. The placebo arm received 1 capsule daily containing 435 mg of maltodextrin and 10 mg of magnesium stearate, thereby providing no polyphenols. The total intake of polyphenols in the active group was 4 mg/day, based on the total phenolic content of FBE as determined using the Folin–Ciocalteu method.

To standardize their cosmetic habits during the study period, all participants were supplied with a base cream to be applied twice a day (morning and evening). The ingredients list (INCI EU) of the base cream was as follows: AQUA, ETHYLHEXYL METHOXYCINNAMATE, PEG-6 STEARATE, ETHYLHEXYL SALICYLATE, BUTYL METHOXYDIBENZOYL METHANE, METHYLENE BIS-BENZOTRIAZOLYL TETRAMETHYLBUTYLPHENOL (NANO), OCTOCRYLENE, TRIOLEIN, GLYCERYL STEARATE, GLYCOL STEARATE, PEG-32 STEARATE, GLYCERYL DIOLEATE, CETYL PALMITATE, DECYL GLUCOSIDE, XANTHAN GUM, PROPYLENE GLYCOL, HYDROXYETHYL ACRYLATE/SODIUM ACRYLOYLDIMETHYL TAURATE COPOLYMER, POLYISOBUTENE, PEG-7 TRIMETHYLOLPROPANE COCONUT ETHER, DISODIUM EDTA, ETHYLHEXYLGLYCERIN, CAPRYLYL GLYCOL, and O-CYMEN-5-OL.

Participants were assigned to receive the active or the placebo product following a computer-generated (PASS 11, version 11.0.8, PASS, LLC, Kaysville, UT, USA) restricted and balanced (1:1 ratio) randomization list. The randomization list was created using the “Wey’s urn” algorithm. Both the FBE and the PL products were identical in size and shape and were numbered. The randomization list was concealed in sequentially numbered, sealed, opaque envelopes. The study adhered to established procedures to maintain separation between the investigator and their collaborators and the staff that delivered the intervention.

### 2.3. Participants and Compliance to Treatment

Eligible participants were all adult Caucasian women aged between 35 and 65 years old (±2 years) with phototypes from I to III, visible “crow’s feet” wrinkles (score ≥ 2 according to the Skin Aging Atlas) [39], mild-to-moderate skin slackness (grades 1–3—internal clinical scale) and uneven skin tone (grades 1–3—internal clinical scale). Exclusion criteria were concomitant participation in other clinical trials, positive anamnesis for pathologies or pharmacological treatment that can potentially interfere with or is incompatible with the test product, change in their routine and lifestyle, exposure to both natural and artificial UV sources, subjects who are accustomed to using tanning beds, topic or oral treatment with products with whitening efficacy 4 months before the screening visit, pregnancy, or breastfeeding. The complete inclusion and exclusion list is reported in the Supplementary Materials (Table S1).

Throughout the study period, participants were asked to write a journal about their food and drink consumption to ensure the stability of their alimentary habits. They were also asked to report the appearance of any adverse events. The compliance with treatment was assessed by counting and recording the remaining pills in each bottle after 28, 56, and 84 days of treatment. The threshold value for compliance with treatment was set at ≥80%.

### 2.4. Primary and Secondary Efficacy Endpoints and Outcome Measures

The primary endpoint was the assessment of product efficacy on the clinical signs of skin aging by the measurement of parameters related to skin texture (wrinkle depth and skin smoothness/roughness) and elasticity.

The secondary endpoint was the assessment of product efficacy in improving the skin complexion, the skin antioxidant capacity, and the tolerability of product use.

The outcome measures were taken in standard temperature (T = 22 ± 4 °C) and humidity (RH = 50 ± 10%) conditions after an acclimation period of 15–20 min to T/HR ambient conditions.

#### 2.4.1. Skin Texture

Wrinkle depth, skin smoothness (Ra), and skin roughness (Rz) were measured in the “crow’s feet” wrinkle area using a Primos^CR^ small-field camera (Canfield Scientific Europe, BV, Utrecht, The Netherlands). Primos^CR^ is a 3D camera based on the fringe projection. The field of view of the camera is 45 × 30 × 25 mm (L × W × H), while the resolution is 20 × 20 × 2 μm (X, Y, Z). The wrinkle depth measurement represents the depth of the deepest wrinkle in the measurement area. The Ra parameter was calculated as the average of the absolute values of profile heights of the roughness profile (peaks and valleys) within the measurement area. The Rz parameter was calculated as the average of the single roughness depths (difference from the highest profile peak to the deepest profile valley) within the measurement area.

The “crow’s feet” wrinkle appearance was also scored by the dermatologist according to the six-point photographic scoring system reported in the Skin Aging Atlas [39], considering the depth of the deepest wrinkle and not considering the number and the length of the wrinkles, as recommended by the authors. 

#### 2.4.2. Skin Elasticity

The skin elasticity was measured in the cheek area using a Cutometer^®^ MPA 580 (Courage+Khazaka, electronic GmbH, Cologne, Germany). The device measures the depth of suction of the skin inside the probe and its release. In this study, we measured the maximum depth of skin penetration inside the probe (R0 parameter, skin firmness) and the ratio between the skin’s residual deformation (Ua) and the maximum elongation (Uf) (R2 parameter, overall skin elasticity). 

The skin firmness was also scored by the dermatologist according to the following scale: 1—not firm skin (inelastic skin characterized by a strong loss of tone, thinned, empty, and not dense skin, the underlying tissues being clearly relaxed, and poor resistance to pinching and pulling, as well as poor elastic recovery after traction), 2—not very firm skin (poorly elastic skin characterized by an evident loss of tone, thinned and less dense skin in some areas, underlying tissues starting to relax, and poor resistance to pinching and pulling, as well as poor elastic recovery after traction), 3—sufficiently firm skin (sufficiently elastic skin characterized by a medium tone, sufficiently full, plump and dense skin, underlying tissues appearing slightly relaxed, sufficient resistance to pinching and pulling with quite good elastic recovery after traction), 4—firm skin (full and plump skin, the underlying tissue does not appear relaxed, and good resistance to pinching and pulling, with good elastic recovery after traction), 5—very firm skin (skin appears full and plump, the underlying tissues do not appear relaxed, and excellent resistance to pinching and pulling; the elastic recovery after traction is excellent).

#### 2.4.3. Skin Complexion

The skin complexion characteristics were measured using a colorimeter/spectrophotometer, CM-700D (Konica Minolta, Tokyo, Japan). The L* (skin lightness) and b* (yellow chroma) parameters were measured to calculate the individual typology angle (ITA°), while the a* (red chroma) parameter was measured with a colorimetric image analysis technique to evaluate the skin’s redness. ITA° was calculated according to the following formula:

(1) $$ITA{}^{\circ}=\tan^{-1}\frac{\left(L^{*}-50\right)}{b^{*}}\times \frac{180}{\pi }.$$

L* and b* parameters for the calculation of ITA° were taken from all over the face (forehead and right/left cheek). 

Improvement in the skin’s complexion evenness was scored by the dermatologist as follows: 1—no variation, 2—slight improvement, 3—moderate improvement, 4—remarkable improvement.

#### 2.4.4. Skin Antioxidant Capacity

The skin antioxidant capacity was measured on skin samples (cheeks) taken with Corneofix^®^ foils (Courage+Khazaka Electronic, Cologne, Germany). The technique allows the collection of different layers of the *stratum corneum*. Five strippings were collected under standardized pressure conditions. The first four strippings were discharged, while stripping no. 5 was collected and stored at −80 °C for a ferric reducing antioxidant power (FRAP) assay, as described by Benzie and Strain [40] and modified by Nobile et al. [24]. Antioxidant capacity is expressed in µmol Fe^II^ per skin sample.

#### 2.4.5. Self-Assessment Questionnaire

At the end of the study, subjects were asked to give their opinion on the tested product by answering a questionnaire about the product’s perceived effects and satisfaction. The questionnaire was taken before any outcome measurement so as not to influence the participants’ answers. Possible answers were “completely agree”, “agree”, “disagree”, or “completely disagree”. “Completely agree” and “agree” were considered in the calculation of the responders. 

### 2.5. Statistical Analysis

Since one volunteer in the FBE group dropped out, the results reported in this paper are for the per-protocol (PP) population and included all the randomized subjects who completed the study.

Means and standard errors of the mean (SEM) were calculated for each group, on each parameter, at each time point. Percentages of variation vs. D0 were also calculated for each time of measurement and each subject, then the mean and SEM of these percentages were calculated for each group.

Intra-group statistical comparisons were performed on raw data using a one-way repeated measures analysis of variance (RM-ANOVA), followed by a post hoc Dunnett’s test for multiple comparisons vs. the baseline (D0) when data followed a normal distribution. Otherwise, the Friedman test was applied, followed by Dunn’s post hoc test for pairwise multiple comparisons of the ranked data. An intra-group comparison of skin antioxidant potential between D0 and D84 was performed using a Wilcoxon matched-pair signed rank test. Inter-group statistical comparisons were performed on the percentage of variation vs. D0, using a one-way analysis of variance (ANOVA), followed by a post hoc Šidák’s correction for multiple comparisons when the data were normally distributed. Otherwise, the Kruskal–Wallis test was applied, followed by Dunn’s post hoc test for pairwise comparisons. The inter-group comparison of skin antioxidant potential and the number of participants expressing positive responses in the self-assessment questionnaire was respectively assessed using Welch’s t test and Fisher’s exact *t*-test. 

Data were considered statistically significant when the *p*-value was <0.05. All statistical analyses were performed using GraphPad Prism 9.5.0 software (GraphPad Software, Boston, MA, USA).

## 3. Results

### 3.1. Participant Characteristics, Tolerability, and Compliance with Treatment

**Figure 1:**
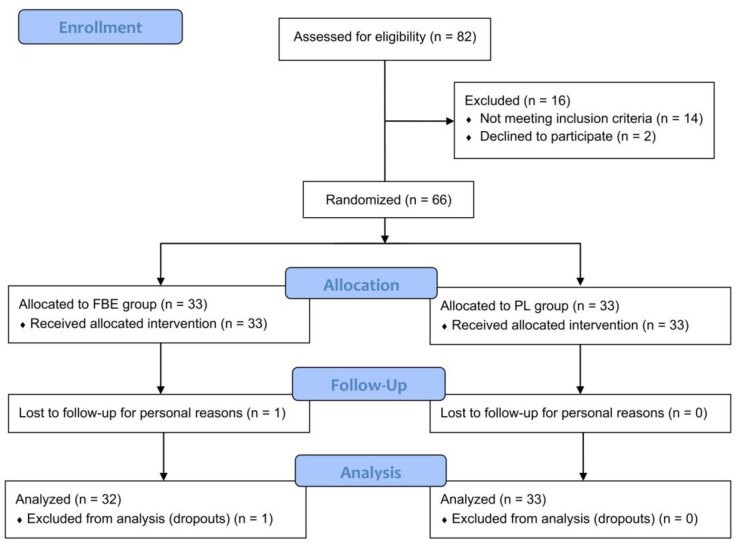
Participants flow diagram.

The study screened 82 subjects (n = 82), 16 of whom were excluded for the following reasons: 14 did not meet the inclusion criteria and 2 declined to participate. The study successfully randomized 66 (n = 66) subjects, who were randomized into the FBE (n = 33) and PL (n = 33) groups. The PP population consisted of 65 subjects since 1 subject in the FBE group dropped out. The reason for the drop-out was related to personal reasons not related to product (cosmetic and food supplement) use. The participant flow chart is shown in Figure 1.

The study enrolled Caucasian (phototype between I and III) women in an age range between 34 and 67 years old (FBE: 51.4 ± 1.4; PL: 52.5 ± 1.3), with visible “crow’s feet” wrinkles (FBE score: 3.4 ± 0.1; PL score: 3.7 ± 0.1). Additional demographics and the baseline characteristics of the participants are shown in Table 1 and clearly indicate the homogeneity of the FBE and PL groups. 

Both FBE and PL products were well tolerated. No adverse effects were reported by the investigator during the entire study period. The overall tolerability was confirmed by 100% of the participants. The alimentary habits were unchanged during the study period and did not represent a covariate between the groups.

Compliance with treatment was 98.7% (min. 95%, max. 100%) in the FBE group and 99.0% in the PL group (min. 91%, max. 100%). 

### 3.2. Primary Endpoints

The wrinkle depth at baseline was 299.9 ± 20.7 μm in the active group and 318.9 ± 14.9 μm in the placebo group. In the active group, this parameter was reduced, as soon as after the first 28 days of treatment, by −3.8% (289.1 ± 20.5, *p* < 0.01), and further reduced by −7.7% (277.6 ± 20.3, *p* < 0.001), and −10.6% (268.9 ± 20.3, *p* < 0.001) after 56 and 84 days of intake, respectively. In the placebo treatment arm, a slight variation of this parameter by −2.1% (312.6 ± 15.7, *p* < 0.05) was observed only after 84 days of product intake. The variation of the wrinkle depth between the FBE and PL groups was statistically significant at all the time points (Table 2). 

A similar trend was seen for skin smoothness (Ra) and skin roughness (Rz). The decrease in both Ra and Rz started from D28 and was further reduced at D56 and D84. In the FBE group, Ra was significantly reduced by −4.6% (*p* < 0.01), −7.5% (*p* < 0.001), and −7.9% (*p* < 0.001), while Rz was reduced by −2.4% (*p* < 0.01), −5.1% (*p* < 0.001), and −7.3% (*p* < 0.001) after 28, 56, and 84 days of product intake, respectively. A slight reduction in Ra (−1.3%, *p* < 0.05) and Rz (−1.5%, *p* < 0.05) was observed in the placebo group, but only at D84. The variation of both Ra and Rz between the FBE and PL groups was statistically significant at all the time points (Table 2). 

An improvement in the “crow’s feet” wrinkles (dermatologist’s scoring) was observed in 31% (−4.5% vs. D0, *p* > 0.05 vs. placebo), 53% (−9.1% vs. D0, *p* < 0.001 vs. placebo), and 56% (−11.7% vs. D0, *p* < 0.001 vs. placebo) of the subjects at D28, D56, and D84 in the FBE group. The maximum number of subjects showing improvement in the PL group was 9% (−1.4% vs. D0) at D84.

**Figure 2:**
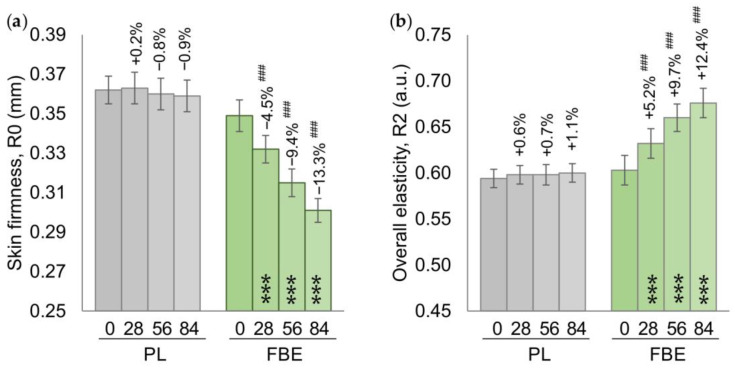
(**a**) Skin firmness (R0). (**b**) Skin overall elasticity. Data are mean ± SEM. The intragroup (vs. baseline) statistical analysis is denoted inside the bars by the symbol *, while the intergroup (active vs. placebo) statistical analysis is denoted above the bars by the symbol #, as follows: ***/### *p* < 0.001.

In the FBE group, both the skin firmness and the overall skin elasticity were improved as soon as after the first 28 days of treatment, and further improved over the duration of treatment. The decrease in the R0 parameter (skin firmness) was by −4.5% (*p* < 0.001), −9.4% (*p* < 0.001), and −13.3% (*p* < 0.001) after 28, 56, and 84 days of product intake, respectively, and can be correlated with an improvement in skin firmness. This improvement was also confirmed by an increase in the R2 parameter (overall skin elasticity) by +5.2% (*p* < 0.001), +9.7% (*p* < 0.001), and +12.4% (*p* < 0.001) after 28, 56, and 84 days of product intake, respectively. In the PL group, both the R0 and the R2 parameters were unchanged. The variation between the FBE and PL groups for both parameters was statistically significant at all the time points (Figure 2).

The skin firmness had clinically improved (dermatologist’s scoring) in 56% (+26.6% vs. D0, *p* < 0.001 vs. placebo), 72% (+32.8% vs. D0, *p* < 0.001 vs. placebo), and 81% (+37.0% vs. D0, *p* < 0.001 vs. placebo) of the subjects in the FBE group at D28, D56, and D84, respectively. The maximum number of subjects showing improvement in the PL group was 9% at D84 (+4.0% vs. D0).

### 3.3. Secondary Endpoints

Skin lightness (L*) was significantly increased in the FBE group at all the time points by +1.2% (*p* < 0.001), +2.2% (*p* < 0.001), and +2.2% (*p* < 0.001) after 28, 56, and 84 days of intake, respectively. This increase in the skin lightness was correlated with an increase (skin whitening effect and decrease in hyperpigmentation) in the ITA° parameter by +9.0% (*p* < 0.001), +16.4% (*p* < 0.001), and +20.8% (*p* < 0.001) after 28, 56, and 84 days of intake, respectively. No variation in the yellowish component (b*) was observed between the FBE and PL groups (Supplementary Table S2).

**Figure 3:**
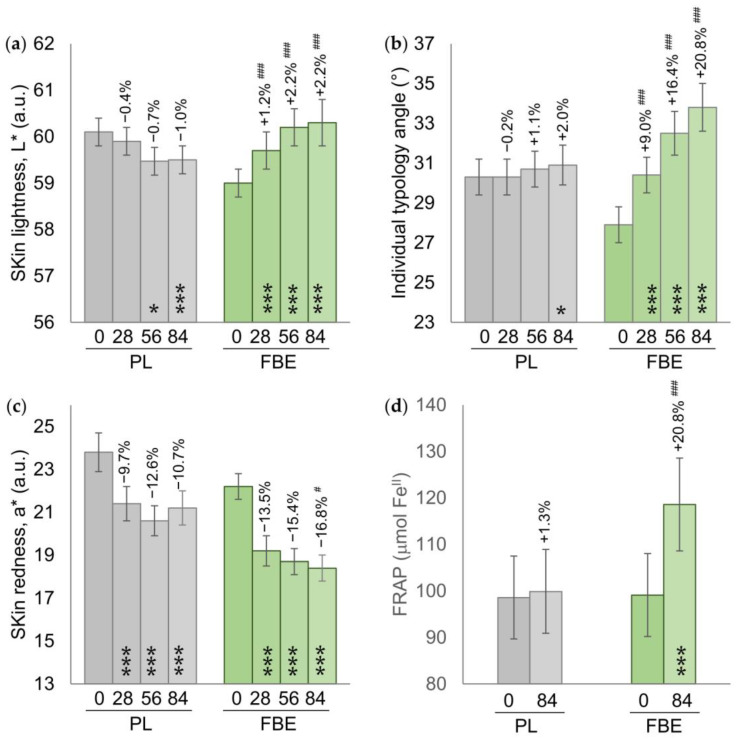
(**a**) Skin lightness (L*). (**b**) Skin pigmentation (ITA°). (**c**) Skin redness (a*). (**d**) Skin antioxidant capacity. Data are mean ± SEM. The intragroup (vs. baseline) statistical analysis is denoted inside the bars by the symbol *, while the intergroup (active vs. placebo) statistical analysis is denoted above the bars by the symbol #, as follows: */# *p* < 0.05, ***/### *p* < 0.001.

In the PL group, L* had decreased (lightening effect) by −0.7% at D56 (*p* < 0.05) and by −1.0% at D84 (*p* < 0.001), while ITA° had slightly increased at D84 (+2.0%, *p* < 0.05). The variation between the FBE and PL groups for both parameters was statistically significant at all the time points (Figure 3a,b). 

The skin redness (a*) had decreased in both the FBE and PL groups. The variation trend was higher in the FBE group compared with the PL group. The a* variation between the FBE and PL groups was statistically significant (*p* < 0.05) at D84 (−16.8% FBE vs. −10.7% PL) (Figure 3c).

An improvement in the skin complexion evenness (dermatologist’s scoring) was observed in 59% (1.7 ± 0.1, *p* < 0.001 vs. placebo), 78% (1.9 ± 0.1, *p* < 0.001 vs. placebo), and 78% (1.9 ± 0.1, *p* < 0.001 vs. placebo) of the subjects of the FBE group at D28, D56, and D84 respectively. The maximum number of subjects recorded as improved in the PL group was 36% at D84 (1.4 ± 0.1).

The skin antioxidant capacity was 99.1 ± 8.9 μmol Fe^II^ in the FBE group and 98.6 ± 8.8 μmol Fe^II^ in the PL group at baseline. In the FBE group, the skin antioxidant capacity had increased by +20.8% (118.6 ± 10.0, *p* < 0.001) at D84, while it was unchanged in the placebo group. The variation between the FBE and PL groups was statistically significant (*p* < 0.001) (Figure 3d).

A post hoc analysis carried out on the postmenopausal subpopulation demonstrated a similar variation in all endpoints in comparison with the whole cohort. The variations between the FBE and the PL groups were statistically significant, as shown in the Supplementary Materials (Table S2).

### 3.4. Self-Assessment Questionnaire

The FBE product was scored as more effective than the PL product (Table 3). Participants from FBE groups perceived a reduced appearance of fine (93.8%) and deep (78.1%) wrinkles, a decrease in the skin’s aging signs (78.1%) with a smoother skin perception (96.9%), an improved complexion (96.9%) giving the skin a healthier glow (90.6%), and less redness (96.9%). In the PL group, the number of subjects giving positive answers was lower (between 54.5% and 72.7%).

## 4. Discussion

Skin aging is a complex and multifactorial process in which cellular senescence is driven by oxidative stress [5,41,42]. The increased oxidative stress caused by exposure to both endogenous and exogenous factors initiates the events of the aging cascade and has a negative impact on extracellular matrix components of the dermis (e.g., metalloproteinases activation, collagen breakdown, protein and DNA damage, etc.) [43,44]. Oxidative stress, and the resulting DNA damage in skin cells, trigger cellular signals that induce the heterogenous overproduction of melanin [42]. Moreover, skin aging and the associated cellular senescence have recently been reported to be influenced by the gut microbiota through the gut–skin axis [45]. From a clinical point of view, these cellular and molecular alterations of the skin are evidenced by increased wrinkling, skin sagging due to a loss of skin elasticity and firmness, and an uneven complexion [46]. 

In the scientific literature, there is increasing evidence of the role of a balanced diet and of food-derived bioactive compounds in promoting and maintaining proper functioning of the skin [1,47]. In particular, polyphenol-rich food supplements have been shown to exert a beneficial effect on skin aging, especially through their well-known antioxidant and anti-inflammatory properties [23,24,25,26,27]. 

In this context, the present study aimed at evaluating the skin antiaging efficacy of a natural extract obtained from the fermentation of wild bilberry fruits with wine yeast (*Saccharomyces cerevisiae*), which contains proanthocyanidins, anthocyanins, and hydroxycinnamic acids. Bilberry fruit is well-known as a rich source of polyphenols, with several reported health benefits [34,48]. However, the bioactivity of berry polyphenols has been questioned in the literature due to their poor bioavailability in the gastrointestinal tract [35,36]. Polyphenols are extensively metabolized by the gut microbiota into bioavailable low-molecular-weight phenolic metabolites, which are assumed to be responsible for the beneficial effects observed following the consumption of polyphenol-containing foods [35]. Fermentation has been reported to improve both the bioavailability of berry bioactive compounds and their subsequent functionality and potential health benefits [36,37]. The additional health benefits brought by the fermentation process may result from both the increase in berry-derived metabolites and the production of bioactive postbiotics. In particular, yeast fermentation was previously demonstrated to be efficient in improving the total phenolic content and the associated antioxidant activity of plant-based foods [37]. Furthermore, FBE was previously demonstrated to exert beneficial effects on atherosclerosis in comparison with its non-fermented counterpart [49], pointing out the advantages of fermentation. The fate of proanthocyanidins during the fermentation process has not been fully elucidated, as these highly polymerized compounds exhibit low stability and are susceptible to condensation [50]. However, it is widely acknowledged that interactions take place between the carbocations produced by the cleavage of proanthocyanidin interflavanic bonds and anthocyanins during wine maturation and aging [51,52], leading to the formation of complex polymeric structures, mainly including pyranoanthocyanins [53]. Consequently, the concentration of free anthocyanidins declines, which was also reported following blueberry and blackberry fermentation [36,54,55]. A similar condensation mechanism between proanthocyanidins and anthocyanins is believed to occur during bilberry fermentation, as free anthocyanins were found to be very low in FBE (<1%). Further phytochemical characterization of FBE, involving high-resolution mass spectrometry, will be necessary to decipher the high complexity of chemical compounds resulting from bilberry fermentation.

The present study showed that FBE intake is effective in improving age-related skin issues. The efficacy of the product was statistically significant when compared to the PL product for all the outcomes and was measured both instrumentally and visually. The improvement in skin texture and in the skin elasticity-related parameters underlies a youthful skin appearance, as demonstrated by the improvement in the wrinkle depth appearance in the “crow’s feet” area. This improvement was not only measured by the instruments or assessed by the dermatologist but was also perceived by most of the study participants, who scored positively those questionnaire items related to visual signs of skin aging (particularly a reduction in the appearance of fine lines and the firmer and softer appearance of the skin). The additional benefits of FBE intake were improvement in the skin complexion-related parameters (i.e., skin pigmentation and skin lightness) and in the redness component of skin color. The beneficial effect of FBE on skin complexion that was observed in this study is in line with a previous pilot study, wherein a significant improvement in skin tone evenness was observed in women with dull skin tone (data not published). Moreover, a similar beneficial effect of FBE on skin texture, firmness/elasticity, and complexion was observed in the population of postmenopausal women, in comparison with the overall study population.

The beneficial role of the polyphenolic components of bilberry extracts in decreasing oxidative stress in different pathological conditions and in different areas of the body is well-known in the literature [34,48,56,57,58,59]. In our study, we demonstrated a strong increase in the skin’s antioxidant capacity, confirming the good bioavailability of FBE bioactive compounds and their delivery at the skin level. Maintaining low levels of reactive oxygen species (ROS) and very low levels of pro-inflammatory-cytokines (either alone or in combination) is necessary to prevent the inflammation associated with aging (inflammaging) [60]. Previous in vitro and in vivo studies on humans have reported the positive effect of bilberry and its extracts in reducing inflammation by downregulating the expression of pro-inflammatory cytokines and enzymes, modulating the signaling pathways, and reducing ROS levels [29]. These findings can be correlated with the decrease in the redness component of the skin that was observed in the present study. 

Anthocyanins have previously been reported as potentially effective compounds to protect the skin from oxidative stress and inflammation, although further clinical validation is still needed [61]. Additionally, proanthocyanidin-rich food extracts have already demonstrated beneficial effects on skin pigmentation [62,63,64,65]. For example, a French maritime pine bark extract was demonstrated to reduce skin photoaging and the pigmentation of age spots in women with mild to moderate photoaging [63], while apple proanthocyanidins have been shown to alleviate the skin pigmentation induced by UV in healthy women [65]. Moreover, polyphenols, including anthocyanins and proanthocyanidins, can positively impact skin health through positive interactions with gut microbiota [66,67]. 

## 5. Conclusions

Our results clearly demonstrated the efficacy of oral supplementation with fermented bilberry extract in reducing the signs of skin aging and in improving skin firmness and complexion, not only in the general population but also in postmenopausal women. Thus, oral supplementation with FBE can be useful as part of a skin antiaging strategy, to fill the gap between the life span and the health span and to make the skin appear to be aging well. 

The putative mechanism underlying decreases in the signs of skin aging can be related to an increase in the skin’s antioxidant capacity and to a decrease in skin inflammation. However, the possible role of FBE in decreasing skin inflammaging and/or influencing the gut–skin axis needs to be investigated further. 

To the best of our knowledge, this is the first study demonstrating the efficacy of FBE intake in improving skin health.

## Figures and Tables

**Table 1:** Demographics and baseline characteristics of study participants.

	PL (n = 33)	FBE (n = 32)	Units
Age	52.5 ± 1.3	51.4 ± 1.4	Years
Postmenopausal population	57.6% (19)	62.5% (20)	% (no.)
Phototype			
I	0.0% (0)	3.1% (1)	% (no.)
II	36.4% (12)	34.4% (11)	% (no.)
III	63.6% (21)	62.5% (20)	% (no.)
Wrinkles scoring	3.7 ± 0.1	3.4 ± 0.1	score
Skin texture			
Wrinkle depth	318.9 ± 14.9	299.9 ± 20.7	μm
Skin smoothness (Ra)	34.9 ± 1.2	34.5 ± 1.2	μm
Skin roughness (Rz)	247.1 ± 10.2	245.4 ± 9.5	μm
Skin elasticity			
R0 (Uf)	0.362 ± 0.007	0.349 ± 0.008	mm
R2 (Ua/Uf)	0.594 ± 0.010	0.603 ± 0.016	a.u.
Skin coloration			
ITA°	30.3 ± 0.9	27.9 ± 0.9	°
L*	60.1 ± 0.3	59.0 ± 0.3	a.u.
b*	17.4 ± 0.3	16.8 ± 0.3	a.u.
a*	23.8 ± 0.9	22.2 ± 0.6	a.u.
Skin antioxidant capacity (FRAP)	98.6 ± 8.8	99.1 ± 8.9	μmol Fe^II^

Continuous data are expressed as mean ± SEM; categorical data are expressed as counts and percentages.

**Table 2:** Skin texture parameters. Data are mean ± SEM. The intragroup (vs. baseline) statistical analysis is denoted by the symbol *, while the intergroup (FBE vs. PL) statistical analysis is denoted by the symbol #, as follows: * *p* < 0.05, **/## *p* < 0.01, ***/### *p* < 0.001.

	D0	D28	D56	D84
Wrinkle depth (mm)				
PL (n = 33)	318.9 ± 14.9	319.5 ± 14.7 (+0.4%)	315.7 ± 15.0 (−1.0%)	312.6 ± 15.7 * (−2.1%)
FBE (n = 32)	299.9 ± 20.7	289.1 ± 20.5 ** (−3.8%) ^##^	277.6 ± 20.3 *** (−7.7%) ^###^	268.9 ± 20.3 *** (−10.6%) ^###^
Skin smoothness, Ra (mm)				
PL (n = 33)	34.9 ± 1.2	35.0 ± 1.2 (+0.4)	34.6 ± 1.2 (−0.8%)	34.4 ± 1.2 * (−1.3%)
FBE (n = 32)	34.5 ± 1.2	33.0 ± 1.3 * (−4.6%) ^##^	32.0 ± 1.2 *** (−7.5%) ^###^	32.0 ± 1.5 *** (−7.9%) ^###^
Skin roughness, Rz (mm)				
PL (n = 33)	247.1 ± 10.2	248.2 ± 10.2 (+0.6%)	245.3 ± 10.4 (−0.8%)	243.4 ± 10.3 * (−1.5%)
FBE (n = 32)	245.4 ± 9.5	239.9 ± 9.7 ** (−2.4%) ^##^	233.5 ± 9.6 *** (−5.1%) ^###^	228.1 ± 9.5 *** (−7.3%) ^###^

**Table 3:** Self-assessment questionnaire. The data given show the percentage of positive answers. The intergroup (FBE vs. PL) statistical analysis is denoted by the symbol #, as follows: # *p* < 0.05, ## *p* < 0.01, ### *p* < 0.001.

You Feel …	PL (n = 33)	FBE (n = 32)
that your skin complexion is improved	72.7%	96.9% ^##^
that your skin has less redness	66.7%	96.9% ^##^
that your skin is brighter/more luminous	66.7%	87.5% ^##^
that your skin has a healthier glow	69.7%	90.6%
that your skin has fewer imperfections	66.7%	87.5%
that your skin is more plumped	66.7%	93.8% ^#^
that your skin is softer	78.8%	100.0% ^##^
that your skin is smoother	69.7%	96.9% ^##^
that your skin is firmer	66.7%	90.6% ^#^
that product reduced the appearance of fine wrinkles	63.6%	93.8% ^#^
that the product reduced the appearance of deep wrinkles	54.5%	78.1%
that the signs of skin aging seem less visible	63.6%	78.1%
that your skin looks visibly younger	63.6%	75.0%
that your skin seems healthier	75.8%	90.6%
that your skin is more moisturized	69.7%	100.0% ^###^
better about yourself in your skin	69.7%	90.6%
that your skin is more beautiful	72.7%	93.8% ^#^
that your overall skin appearance is improved	69.7%	96.9% ^##^
Was the treatment well tolerated?	100.0%	100.0%
Are you globally satisfied with the efficacy of the product?	75.8%	96.9% ^#^

## Data Availability

The data presented in this study are available on request from the corresponding author. The data are not publicly available since they are the property of the sponsor of the study (Seppic, France).

## Supplementary Materials

The following supporting information can be downloaded at: https://www.mdpi.com/article/10.3390/nu16142203/s1, Table S1. Inclusion and exclusion criteria. Table S2. Skin yellowness (b*). Data are mean ± SEM. Table S3. Variation of primary and secondary endpoints in a population of postmenopausal women.
