# Supplementary material for: Antiaging, Brightening, and Antioxidant Efficacy of Fermented Bilberry Extract (Vaccinium myrtillus): A Randomized, Double-Blind, Placebo-Controlled Trial
Source: Nutrients. 2024 Jul 10;16(14):2203. doi: 10.3390/nu16142203 (PMC11280171; doi:10.3390/nu16142203)
Supplement: Supplementary file 1 [file nutrients-16-02203-s001.zip › nutrients-3069358-supplementary.pdf]

SUPPLEMENTARY INFORMATION

Antiaging, Brightening and Antioxidant Efficacy of Fermented Bilberry Extract (*Vaccinium myrtillus*): a randomized, double-blind, placebo-controlled trial

Vincenzo Nobile, Stéphanie Dudonné, Catherine Kern, Gloria Roveda, and Christine Garcia

Supplementary Table S1. Inclusion and exclusion criteria.

No.	Inclusion criteria
01	Healthy female subjects,
02	Caucasian ethnicity,
03	Age between 35 and 65 (limits of ±2 years old could be included)
04	Phototypes from I to III
05	Subject with uneven skin tone (grade 1-3 – internal clinical scale)
06	Subject with visible Crow's feet wrinkles (≥2 according to Skin Aging Atlas – Caucasian and Asian Type - Bazin Roland)
07	Subject with mild-moderate skin slackness at cheek level (grade 1-3 – internal clinical scale).
08	Willingness to not assume during the study period products other than the test product
09	Subjects registered with National Health Service (NHS)
10	Subjects certifying the truthfulness of the personal data disclosed to the investigator
11	Subjects able to understand the language used in the investigation centre and the information given by the investigator
12	Subjects able to respect the instructions given by the investigator as well as able to respect the study constraints and specific requirements
13	The pharmacological therapy (except for the pharmacological therapy in the non-inclusion criteria) should be stable for at least one month without any changes expected or planned during the study
14	Commitment not to change the daily routine or the lifestyle
15	Subjects who have not been recently involved in any other similar study (at least one month of wash-out)
16	Subject under effective contraception (oral/not oral) therapy
17	Subjects who accept not to expose in intensive way to UV rays during the whole study duration.
18	Subject aware of the study procedures and having signed an informed consent form and privacy information form
**No.**	**Exclusion criteria**
01	Subject does not meet the inclusion criteria,
02	Subjects with acute or chronic diseases able to interfere with the outcome of the study or that are considered dangerous for the subject or incompatible with the study requirements
03	Subjects participating or planning to participate in other clinical trials
04	Subjects deprived of freedom by administrative or legal decision or under guardianship
05	Subjects not able to be contacted in case of emergency
06	Subjects admitted to a health or social facility
07	Subjects planning a hospitalisation during the study
08	Subjects who participated in a similar study without respecting an adequate washout period
09	Subjects having an acute, chronic or progressive illness liable to interfere with the study data or considered by the Investigator hazardous for the subject or incompatible with the study requirements
10	Subjects under pharmacological treatments that are considered incompatible with the study requirement by the investigator
11	Subjects having an acute, chronic or progressive illness liable to interfere with the study data or considered by the Investigator hazardous for the subject or incompatible with the study requirements
12	Subjects having a skin disease or condition liable to interfere with the study data or considered by the Investigator hazardous for the subject or incompatible with the study requirements
13	Subject with known or suspected sensitization to one or more test formulation ingredients
14	Subject breastfeeding, pregnant or not willing to take necessary precautions to avoid pregnancy during the study (for the women of childbearing potential)
15	Consumption of food supplement(s) and/or use of topical skincare products with whitening activity currently or within the past 4 weeks before the study
16	Subjects accustomed to use tanning beds
17	Subjects taking medication with photosensitizing potential, drugs and/or dietary supplements able to induce skin colouring, corticoids, currently or during the month before the study.

Supplementary Table S2. Skin yellowness (b*). Data are mean ± SEM. The intragroup (vs. baseline) statistical analysis is denoted by the symbol *, as follows: ** $p < 0.01$, *** $p < 0.001$.

	D0	D28	D56	D84
PL (n = 33)	17.4 ± 0.3	16.8 ± 0.3 ** (-2.8%)	16.3 ± 0.3 *** (−5.7%)	15.8 ± 0.2 *** (−8.8%)
FBE (n = 32)	16.8 ± 0.3	16.4 ± 0.3 (−2.3%)	15.9 ± 0.3 *** (−5.2%)	15.1 ± 0.3 *** (−9.5%)

Supplementary Table S3. Variation of primary and secondary endpoints in the postmenopausal women population. Data are mean ± SEM. The intergroup (FBE *vs.* PL) statistical analysis is reported by the symbol #, as follows: # $p < 0.05$, ## $p < 0.01$, ### $p < 0.001$. n.d. not determined.

	**PL (n = 19)**			**FBE (n = 20)**		
	**D28**	**D56**	**D84**	**D28**	**D56**	**D84**
Wrinkle depth	–0.3%	–1.9%	–3.1%	–4.0%	–7.9%##	–10.1%###
Skin smoothness (Ra)	–0.3%	–1.1%	–2.2%	–4.3%#	–7.1%###	–8.6%###
Skin roughness (Rz)	+0.2%	–1.1%	–2.0%	–2.1%	–5.0%#	–7.4%###
Skin distensibility (R0)	+0.5%	–0.5%	–0.5%	–3.7%#	–7.7%###	–11.7%###
Skin overall elasticity (R2)	+0.3%	+0.3%	+1.2%	+5.1%##	+9.4%###	+11.5%###
Skin lightness (L*)	–0.4%	–0.4%	–0.6%	+1.1%##	+2.1%###	+2.1%###
Skin pigmentation (ITA°)	–1.2%	+1.3%	+2.0%	+8.3%###	+16.2%###	+20.8%###
Skin redness (a*)	–8.7%	–11.0%	–8.0%	–13.8%	–15.3%	–16.0%#
Skin antioxidant capacity (FRAP)	n.d.	n.d.	+1.5%	n.d.	n.d.	+19.7%###
